# Comparison of survival and clinicopathologic features in colorectal cancer among African American, Caucasian, and Chinese patients treated in the United States: Results from the surveillance epidemiology and end results (SEER) database

**DOI:** 10.18632/oncotarget.5223

**Published:** 2015-08-24

**Authors:** Junzhong Lin, Miaozhen Qiu, Ruihua Xu, Adrian Sandra Dobs

**Affiliations:** ^1^ Department of Colorectal Surgery, Sun Yat-Sen University Cancer Center, State Key Laboratory of Oncology in South China, Collaborative Innovation Center for Cancer Medicine, Guangzhou, China; ^2^ Department of Medical Oncology, Sun Yat-Sen University Cancer Center, State Key Laboratory of Oncology in South China, Collaborative Innovation Center for Cancer Medicine, Guangzhou, China; ^3^ Department of Oncology, The Sidney Kimmel Comprehensive Cancer Center, The Johns Hopkins University School of Medicine, Baltimore, MD, USA; ^4^ Division of Endocrinology, Diabetes and Metabolism, The Johns Hopkins Center to Reduce Cancer Disparities, The Johns Hopkins University School of Medicine, Baltimore, MD, USA

**Keywords:** colorectal cancer, race/ethnicity, SEER, survival analysis

## Abstract

African American patients of colorectal cancer (CRC) were found to have a worse prognosis than Caucasians, but it has not been fully understood about the survival difference among Chinese and these two races above. In this study, we used the Surveillance, Epidemiology and End Results database to analyze the survival difference among these three race/ethnicities in the United States. Adenocarcinoma patients of colorectal cancer with a race/ethnicity of Caucasian, Chinese and African American were enrolled for study. Patients were excluded if they had more than one primary cancer but the CRC was not the first one, had unknown cause of death or unknown survival months. The 5-year cause specific survival (CSS) was our primary endpoint. Totally, there were 585,670 eligible patients for analysis. Chinese patients had the best and African American patients had the worst 5-year CSS (66.7% vs 55.9%), *P* < 0.001. The 5-year CSS for Caucasian patients was 62.9%. Race/ethnicity was an independent prognostic factor in the multivariate analysis, *P* < 0.001. The comparison of clinicopathologic factors among these three race/ethnicities showed that the insurance coverage rate, income, percentage that completing high school and percentage of urban residence was lowest in the African American patients. Chinese patients had the highest percentage of married, while African American patients ranked lowest. More African American patients were diagnosed as stage IV and had high percentage of signet ring cell and mucinous adenocarcinoma. It is likely that biological differences as well as socioeconomic status both contribute to the survival disparity among the different race/ethnicities.

## INTRODUCTION

Colorectal cancer (CRC) is among the leading causes of cancer related deaths all over the world [1]. The survival of CRC patients differs according to race in the United States. Several studies have shown elevated CRC mortality rate and shorter survival for African Americans compared with Caucasians patients [2, 3]. CRC was also one of the most common cancers in Chinese population. It was reported to have 310,244 newly diagnosed CRC patients in China in 2011 [4]. There were few reports about the survival differences among African American, Caucasian and Chinese CRC patients. In 2012, Hashiguchi Y et al. reported the impact of race/ethnicity on prognosis of colon cancer patients [5]. They showed that East Asian Americanpatients (including Chinese patients) had significantly betterprognosis, andAfrican Americanpatients had worseprognosis than non-Hispanicwhite patients [5]. However, they only analyzed colon cancer patients who had underwent surgery. We are not aware of any other reports comparing African American, Caucasian, and Chinese CRC Patients.

It is hard to get a convincing conclusion if we compare these three groups of patients using different databases. The Surveillance Epidemiology and End Results (SEER) Database (http://seer.cancer.gov/data/citation.html), a report on the most recent cancer incidence, mortality, survival, prevalence, and lifetime risk statistics, is published annually by the Data Analysis and Interpretation Branch of the National Cancer Institute, USA. It provides various race/ethnicities (including white, black and Chinese) with a large sample size in the United States, moreover it contains no identifiers and is therefore a good database for our study.

In this study, we used data from the SEER cancer-registry program of individuals diagnosed with CRC from 1973 to 2012 to compare the survival and clinicopathologic features among African American, Caucasian, and Chinese Patients.

## RESULTS

### Patient baseline characteristics

The study identified 585,670 adenocarcinoma patients of the colorectal cancer. Of these patients, 514,497 (87.8 %) were Caucasian, 61062 (10.4%) were African American and 10111(1.8%) were Chinese. Table 1 showed the basic features among these three groups of patients.

**Table 1:** Basic characteristics among the three race/ethnicities

	Caucasian *N* = 514,497 (%)	Chinese *N* = 10,111 (%)	African American *N* = 61,062 (%)	*P*^1^ value	*P*^2^ value	*P*^3^ value
Sex *N* = 585,670				<0.001	<0.001	<0.001
Male	264,671 (51.4)	5,379 (53.2)	29,131 (47.7)			
Female	249,826 (48.6)	4,732 (46.8)	31,931 (52.3)			
Age (Mean±SD)	68.5±13.1	67.7±13.5	64.6±13.3	<0.001	<0.001	<0.001
Family income*, (Mean±SD)	72,694±17,302	82,104±14,833	65,691±16,293	<0.001	<0.001	<0.001
Education (%)^#^ (Mean±SD)	86.2±5.8	84.8±5.2	84.3±5.2	<0.001	<0.001	<0.001
Married status *N* = 563,047				<0.001	<0.001	<0.001
Single or unmarried	50,681 (10.2)	855 (8.7)	12,995 (22.4)			
Married	293,840 (59.3)	6,845 (69.9)	24,950 (43.0)			
Widowed	108,270 (21.9)	1,695 (17.3)	12,043 (20.7)			
Separated or divorced	42,395 (8.6)	404 (4.1)	8,074 (13.9)			
Residence *N* = 585,670				<0.001	<0.001	<0.001
Rural	42994 (8.4)	79 (0.8)	12944 (21.2)			
Urban	471503 (91.6)	10032 (99.2)	48118 (78.8)			
Location *N* = 585,670				<0.001	<0.001	<0.001
Left colon	209,130 (40.6)	4,800 (47.4)	24,561 (40.2)			
Right colon	200,982 (39.1)	3,202 (31.7)	26,959 (44.2)			
Rectum	104,385 (20.3)	2,109 (20.9)	9,542 (15.6)			
Time of diagnosis *N* = 585,670				<0.001	<0.001	<0.001
1973-1979	48,090 (24.7)	455 (23.6)	3,222 (22.3)			
1980-1989	83,242 (27.3)	1,097 (26.6)	6,986 (24.3)			
1990-1999	101,532 (28.5)	2,616 (31.9)	11,250 (27.4)			
2000-2012	281,633 (19.5)	5,943 (17.9)	39,604 (26.0)			
AJCC 6^th^ TNM stage *N* = 202,931				<0.001	<0.001	<0.001
I	44,713 (25.7)	880 (23.8)	5,811 (22.9)			
II	47,986 (27.6)	991 (26.8)	6,329 (24.9)			
III	48,404 (27.9)	1,153 (31.2)	7,000 (27.5)			
IV	32,722 (18.8)	667 (18.0)	6,275 (24.7)			
Histology subgroup *N* = 585,670				<0.001	0.293	<0.001
Signet Ring cell	4,283 (0.8)	66 (0.6)	481 (0.8)			
Mucinous adenocarcinoma	47,640 (9.3)	756 (7.5)	5,737 (9.4)			
Other adenocarcinoma	462,574 (89.9)	9,289 (91.9)	54,844 (89.8)			
Grade *N* = 500,181				<0.001	<0.001	<0.001
Well differentiated	53,993 (12.3)	757 (8.5)	6,113 (11.7)			
Moderately differentiated	295,400 (67.3)	6,492 (72.9)	37,481 (72.0)			
Poorly differentiated	84,042 (19.1)	1,569 (17.6)	7,951 (15.3)			
Undifferentiated	5,787 (1.3)	83 (1.0)	513 (1.0)			
Insurance status *N* = 139,962				0.683	<0.001	<0.001
Uninsured	3,814 (3.2)	92 (3.3)	1,248 (7.0)			
Insured	115,472 (96.8)	2,666 (96.7)	16,670 (93.0)			

SD: Standard deviation

AJCC: American Joint Committee on Cancer

TNM: Tumor-Node-Metastasis

* USA dollars

# Percent that completed high school

*P*^1^: The comparison between Caucasian and Chinese patients

*P*^2^: The comparison between Caucasian and African-American patients

*P*^3^: The comparison between Chinese and African-American patients

Overall there was 238,491 (40.7%) left colon cancer patients, 231,143 (39.5%) right colon cancer patients and 116,036 (19.8%) rectum cancer patients. Chinese patients had the highest percentage of left colon cancer, African American had more right colon cancer, while Caucasian had the highest percentage of rectum cancer.

African American patients tended to be younger than both Caucasian and Chinese patients (mean age: 64.3 *vs* 68.5 *vs* 67.6, respectively, *P* < 0.001). African American patients showed a higher ratio of female patients (52.3%) while male patients were more common in both Caucasian and Chinese patients (51.4% and 53.2%), *P* < 0.001.

The Tumor-Node-Metastasis (TNM) stage had no significant difference between Caucasian and African American patients, while Chinese patients had a significant lower percentage of stage IV diseases than both Caucasian and African American patients (18.0% *vs* 18.8% *vs* 24.7%, *P* < 0.001). Furthermore, we found that Chinese patients had the lower percentage of signet ring cell and mucinous adenocarcinoma than both Caucasian and African American patients (8.1% *vs* 10.1% *vs* 10.2%, *P* < 0.001).

About 97%% of Caucasian and Chinese patients were covered by the insurance, significantly higher than black patients, 93%, *P* < 0.001. Almost 70% of Chinese patients were married, significantly higher than Caucasian or African American patients (59.3% and 43.0%), *P* < 0.001. African American patients had lower income and higher rural residence than both Caucasian and Chinese patients. As for the education level, Caucasian patients had higher percentage of completing high school.

### Survival

**Figure 1:**
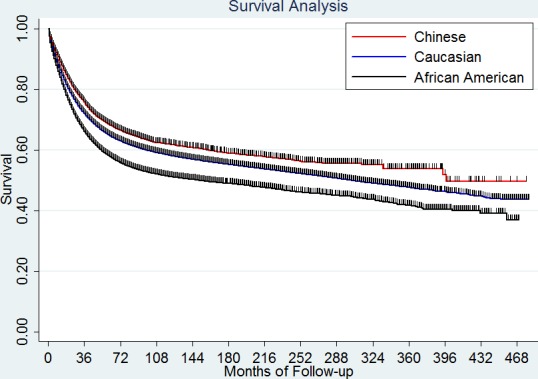
Survival difference among the three race/ethnicities

In this study, 188,682 deaths (36.7 %) were observed among the Caucasian patients (*N* = 514,497), 3305 deaths (32.7 %) among Chinese patients (*N* = 10,111) and 25,214 deaths (41.3%) among the African American patients (*N* = 61,062). The Chinese patients had significantly longer 5-year cause specific survival (CSS) (66.7%) than the Caucasians and African Americans (62.9% and 55.9%), *P* < 0.001 (Figure 1).

The median age of the whole population was 69 years old. We divided the patients into two groups according to the age: < 70 years old (younger patients) and > 69 years old (older patients). The younger patients had a significantly better 5-year CSS than the older patients (65.6% *vs* 58.7%, *P* < 0.001). Female patients had a slightly better 5-year CSS than male (62.5% *vs* 62.1%, *P* = 0.0024). The site of tumor also had an effect on the survival, left colon had better 5-year CSS than right colon and rectum cancer patients, 64.0% *vs* 61.6% *vs* 60.3%, respectively, *P* < 0.001 (Table 2).

**Table 2:** Univariate analysis of 5-year cause specific survival

	5-year CSS (95% CI)	Univariate analysis	
		Log rank	*P* value
Sex		24.38	<0.001
Male	62.1% (61.9%-62.3%)		
Female	62.5% (62.3%-62.7%)		
Age		2697.73	<0.001
<70	65.6% (65.4%-65.8%)		
>69	58.7% (58.5%-58.9%)		
Race/ethnicity		985.98	<0.001
Caucasian	62.9% (62.8%-63.1%)		
Chinese	66.7% (65.7%-67.7%)		
African American	55.9% (55.4%-56.3%)		
Married status		3034.92	<0.001
Single	57.7% (57.3%-58.2%)		
Separated or divorced	59.6% (59.1%-60.0%)		
Widowed	56.6% (56.3%-56.9%)		
Married	65.4% (65.2%-65.5%)		
Primary tumor		674.08	<0.001
Left colon	64.0% (63.7%-64.2%)		
Right colon	61.6% (61.4%-61.8%)		
Rectum	60.3% (60.0%-60.6%)		
AJCC 6^th^ TNM stage		84623.76	<0.001
I	90.9% (90.6%-91.2%)		
II	81.3% (80.9%-81.7%)		
III	66.8% (66.3%-67.3%)		
IV	12.2% (11.8%-12.6%)		
Histology subtype		2769.67	<0.001
Signet ring cell	30.4% (29%-31.9%)		
Mucinous adenocarcinoma	57.1% (56.6%-57.6%)		
Other adenocarcinoma	63.1% (63%-63.3%)		
Grade		13698.5	<0.001
Well differentiated	73.9% (73.5%-74.3%)		
Moderately differentiated	65.8% (65.6%-66%)		
Poorly differentiated	48.1% (47.8%-48.5%)		
Undifferentiated	45.9% (44.5%-47.4%)		
Insurance status		105.08	<0.001
Uninsured	57.8% (55.7%-59.9%)		
Insured	67.2% (66.8%-67.5%)		
Time of diagnosis		4835.37	<0.001
1973-1979	50.7% (50.2%-51.1%)		
1980-1989	57.3% (57.0%-57.7%)		
1990-1999	62.3% (62.0%-62.6%)		
2000-2012	65.9% (65.7%-66.1%)		

CSS: Cause specific survival

CI: Confidence interval

AJCC: American Joint Committee on Cancer

TNM: Tumor-Node-Metastasis

No doubt, the TNM stage was significantly correlated with survival. It was 90.9%, 81.3%, 66.8% and 12.2% for patients from stage I to stage IV respectively, *P* < 0.001. As for the histology subtype, signet ring cells had the worst 5-year CSS than the mucinous adenocarcinoma or other adenocarcinoma, 30.4% *vs* 57.1% *vs* 63.1%, *P* < 0.001. More in detail, when we compared the 5-year CSS in patients with different grades, we found that the survival became poorer as the tumor grade progressed from well to undifferentiated, 73.9% for well differentiated, 65.8% for moderately differentiated, 48.1% for poorly differentiated and 45.9% for undifferentiated tumors, *P* < 0.001.

We also analyzed the influence of insurance on the survival and found that there was 10% higher 5-year CSS in the insured group than uninsured group, 67.2% *vs* 57.8%, *P* < 0.001.

With the change in secular time, we found that the survival of CRC patients became better and better. In 1970's, the 5-year CSS for CRC patients was only 50.7% and it increased to be 65.9% in the 21 century, *P* < 0.001.

### Multivariate analysis

Variables showing a trend for association with survival (*P* < 0.05) were selected in the cox proportional hazards model. Sex, age, race/ethnicity, married status, tumor site, TNM stage, histologic subtypes, grade, insurance status as well as the time of diagnosis were all independent prognostic factors in the multivariable analysis. The HR for race/ethnicity was 1.09, 95% confidence interval (CI): 1.05-1.13 (Table 3), *P* < 0.001. Compared for Caucasian patients, the hazard ratio (HR) for Chinese patients was 0.86 and 1.22 for African American.

**Table 3:** Multivariate analysis of survival

	Hazard ratio	Standard error	*P* value	95% confidence interval
Sex				
Male	Reference			
Female	0.92	0.013	<0.001	0.89-0.95
Age				
<70	Reference			
>69	1.87	0.029	<0.001	1.81-1.93
Race/ethnicity				
White	Reference			
Chinese	0.86	0.046	0.005	0.78-0.96
African American	1.22	0.024	<0.001	1.17-1.27
Married status				
Married	Reference			
Separated/Divorced	1.21	0.027	<0.001	1.16-1.26
Single	1.32	0.026	<0.001	1.27-1.37
Widowed	1.41	0.028	<0.001	1.36-1.47
Site				
Right colon	Reference			
Left colon	0.88	0.014	<0.001	0.85-0.90
Rectum	1.08	0.021	<0.001	1.04-1.13
AJCC 6^th^ TNM stage				
I	Reference			
II	1.81	0.064	<0.001	1.69-1.94
III	3.90	0.128	<0.001	3.66-4.16
IV	21.39	0.681	<0.001	20.10-22.77
Histology subtype				
Other adenocarcinoma	Reference			
Mucinous adenocarcinoma	1,06	0.025	0.014	1.01-1.11
Signet ring cell	1.54	0.073	<0.001	1.40-1.69
Grade				
Well differentiated	Reference			
Moderate differentiated	1.14	0.037	<0.001	1.07-1.22
Poorly differentiated	1.73	0.059	<0.001	1.62-1.85
Undifferentiated	1.82	0.090	<0.001	1.65-2.00
Insurance status				
Insured	Reference			
Uninsured	1.21	0.042	<0.001	1.13-1.30
Time of diagnosis				
1973-1979	Reference			
1980-1989	0.85	0.007	<0.001	0.83-0.86
1990-1999	0.73	0.006	<0.001	0.71-0.74
2000-2012	0.64	0.005	<0.001	0.63-0.65

AJCC: American Joint Committee on Cancer

TNM: Tumor-Node-Metastasis

## DISCUSSION

Previous studies had showed that despite improvements in treatment, racial disparities persisted in CRC incidence, mortality and survival [6-8]. African Americans were reported to have higher incidence rates and lower survival rates for CRC than Caucasian in the United States [2, 3, 9, 10]. According to the Global Cancer Statistics, the incidence of CRC is rising in East Asia including Hong Kong and Shanghai in China [4, 11]. To our best knowledge, this is the first population-based comparisons of CSS in CRC patients with these three race/ethnicities. In the present study, we used all the CRC adenocarcinoma patients for analysis and found that Chinese patients had a significantly better 5-year CSS than the other two races while African American patients had the worst prognosis. Compared with Caucasian patients, the risk of cancer related death was 14% lower in Chinese patients and 22% higher in African American patients. What caused the difference? Numerous reports have indicated that socioeconomic status seemed to be important in the poor prognosis of African American patients [9, 12]. While, other studies suggested that biological factors might be responsible for the poor survival in African American patients [3]. In the present study, we also made a comparison of clinicopathologic features among these three groups. Some of the differences may explain the survival disparity.

We classified the basic characteristics in Table 1 into two groups: socioeconomic factors (including married status, insurance status, income, residence and time of diagnosis) and biologic factors (including age, sex, site, TNM stage, histologic subgroup and grade). As for the socioeconomic factors, we found that the insurance coverage rate, income and urban residence was lowest in the African American patients, moreover, Chinese patients had the highest percentage of married, while African American patients ranked lowest. The insurance status would affect the treatment and furthermore cause a difference in survival [13, 14]. Previous study showed that marital status was an independent prognostic factor for survival in CRC patients [15, 16]. More in detail, unmarried patients were at greater risk of cancer specific mortality [15]. In the present study, we also found that married patients had a better CSS compared with the other subgroups of married status patients. Insured patients also had a better CSS than uninsured patients. It seemed that socioeconomic status might account for the difference in prognosis among these three race/ethnicities.

More African American patients were diagnosed as stage IV and had high percentage of signet ring cell and mucinous adenocarcinoma. Previous studies showed that CRC patients with signet-ring cells or mucinous adenocarcinoma had poor prognoses and were recommended to be given significant clinical attention [17, 18]. Our study also showed that patients with signet ring cell and mucinous adenocarcinoma had significantly poorer 5-year CSS than those with other adenocarcinoma.

More and more evidence suggested that primary colon and rectal tumors should no longer be considered as a single disease entity [19-21]. Previous reports had described differences in biology and outcome based on whether the primary was right or left sided [22, 23]. Poor prognosis of right-sided primary was consistently observed compared with those with a left-sided primary [21, 24]. Our data also showed that left side colon patients had a significantly better 5-year CSS compared with right side and rectum cancer patients. Moreover Chinese patients had a highest proportion of left colon cancer among these three race/ethnicities.

However, we also found some controversial phenomenon. African-America CRC patients were diagnosed at a younger age while young patients were supposed to have a better survival. Moreover, African American CRC patients had higher proportion of well differentiated and lower proportion of undifferentiated tumors than Chinese patients, but they still had a poorer survival than Chinese patients. The multivariate analysis confirmed that these clinicopathologic factors analyzed above were all independent prognostic factors. Until now, we still have no idea how these factors work together and how much they predict outcome. It is likely that biological differences as well as socioeconomic status both contributed to the survival disparity among the different race/ethnicities.

There were few studies about the survival of the whole Chinese CRC patients in China. Most of the studies focus on the subgroups of CRC patients. One study showed that the 5 year survival of Chinese CRC patients in China was 68.0% [25]. Though it was hard to make a comparison across the studies, there is no large difference for Chinese CRC patients in China *vs*. Chinese America. Of note, in this study, we focused on Chinese American CRC patients, while not Asian American CRC patients. Since white, black and Chinese are three independent categories in the item of race/ethnicity of SEER database. If we use Asian data, we have to combine several categories together.

We realized there were some limitations of our analysis. Although this was a population-based registry and we had a large sample size, we were unable to include some other known prognostic factors into analysis such as chemotherapy use, smoking status and aspirin usage, since the database does not have these information. Moreover, not all the patients enrolled for analysis had the full information of all the clinical features. Finally, the genetic difference among these three race/ethnicities are also out of our reach.

In conclusion, we used the SEER database to evaluate the survival disparity of CRC patients with the race/ethnicity of African American, Caucasian and Chinese. We showed that Chinese patients had the best 5-year CSS and African American had the poorest 5-year CSS. The comparison of clinicopathologic features among these three race/ethnicities provided some clues to explain the survival disparity. Both biological differences and socioeconomic status may contribute to the survival disparity.

## MATERIALS AND METHODS

### Database

The SEER database is the largest publicly available cancer dataset. It is a population-based cancer registry across several disparate geographic regions. The SEER research data include cancer incidence and prevalence as well as demographic information tabulated by age, sex, race/ethnicity, year of diagnosis and geographic region. The exact dataset we used for this analysis was Surveillance, Epidemiology, and End Results (SEER) Program (www.seer.cancer.gov) Research Data (1973-2012), National Cancer Institute, DCCPS, Surveillance Research Program, Surveillance Systems Branch, released April 2015, based on the November 2014 submission, “Incidence-SEER 18 Regs Research Data + Hurricane Katrina Impacted Louisiana Cases, Nov 2014 Sub (1973-2012 varying)”

### Outcome variables

The anatomic subsites of the left colon, right colon and rectum were categorized according to the International Classification of Diseases for Oncology, third edition (ICD-0-3) topography codes. Right-sided colon cancers were identified with the following SEER cancer site codes: cecum (ICD-0-3 code C18.0), ascending colon (Code C18.2), hepatic flexure (Code C18.3) and transverse colon (Code C18.4). Left-sided colon cancers were identified with codes: splenic flexure (Code C18.5), descending colon (code C18.6), sigmoid colon (code C18.7) and rectosigmoid (code C19.9). Rectal cancer was identified as code C20.9.

In this manuscript, only adenocarcinoma patients were enrolled (SEER histology codes: signet ring cell, 8490; mucinous adenocarcinoma, 8480 and 8481; other adenocarcinoma: 8140 to 8147, 8210 to 8211, 8220 to 8221, and 8260 to 8263).

For the insurance status, individuals in the “Any Medicaid”, “Insured” and “Insured/No specifics” groups were clustered together as “Insured group”. For the married status, we combined the “Separated” and “Divorced” as “separated / divorced” group, and combined the “Single” and “Unmarried or Domestic Partner” and “Single” group.

### Patient population

The study population was based on the SEER cancer registry. Left colon, right colon as well as rectum adenocarcinoma patients with a race/ethnicity of white, black or Chinese from 1973 through 2012 were eligible for the study. Patients were excluded if they had more than one primary cancer but the CRC wasn't the first one, had unknown cause of death or unknown survival months.

Age, sex, American Joint Committee on Cancer (AJCC) Cancer Staging Manual (6^th^ edition, 2004), histological subtype, grade, insurance status, married status, time of diagnosis, survival time and CSS were extracted from the SEER database.

### Statistical methods

The patients’ demographic and tumor characteristics were summarized with descriptive statistics (Table or Figure). Comparisons of categorical variables among these three race/ethnicity populations were performed using the Chi square test, and continuous variables were compared using Student's t test. The primary endpoint of this study was 5-year CSS, which was calculated from the date of diagnosis to the date of cancer specific death. Deaths attributed to CRC were treated as events and deaths from other causes were treated as censored observations. Survival function estimation and comparison among African American, Caucasian and Chinese were performed using Kaplan-Meier estimates and the log-rank test. The independence of the prognostic effect of race/ethnicity was evaluated by adjusting for other known factors including age at diagnosis and tumor stage. The multivariate Cox proportional hazard model was used to evaluate the HR and the 95 % CI for all the known prognostic factors. All of statistical analyses were performed using the Intercooled Stata 13.0 (Stata Corporation, College Station, TX). Statistical significance was set at two-sided *P* < 0.05.
